# Strengthening of AA5754 Aluminum Alloy by DRECE Process Followed by Annealing Response Investigation

**DOI:** 10.3390/ma13020301

**Published:** 2020-01-10

**Authors:** Przemysław Snopiński, Tomasz Tański, Klaudiusz Gołombek, Stanislav Rusz, Ondřej Hilser, Tibor Donič, Paweł M. Nuckowski, Marcin Benedyk

**Affiliations:** 1 Division of Material Processing Technology, Management and Computer Techniques in Materials Science, Institute of Engineering Materials and Biomaterials, Silesian University of Technology, 44-100 Gliwice, Poland; tomasz.tanski@polsl.pl (T.T.); klaudiusz.golombek@polsl.pl (K.G.); pawel.nuckowski@polsl.pl (P.M.N.); 2 Department of Mechanical Technology, Faculty of Mechanical Engineering, VŠB-Technical University of Ostrava, 17. listopadu 15, 708 33 Ostrava, Czech Republic; stanislav.rusz@vsb.cz (S.R.); ondrej.hilser@vsb.cz (O.H.); 3 Research Centre of University of Zilina, University of Zilina, 010 26 Zilina, Slovak Republic; tibor.donic@rc.uniza.sk; 4 Paks’D Sp z.o.o. Strzelecka 74, 43-100 Tychy, Poland; mbenedyk@paksd.co

**Keywords:** DRECE, XRD, EBSD, structure

## Abstract

In this study, a dual rolls equal channel extrusion (DRECE) process has been applied for improving the mechanical properties of the 5754 alloy. Supplementary experiments involving metallography, electron backscattered diffraction (EBSD), and XRD tests were carried out to evaluate the effect of the DRECE process. XRD analysis showed that the maximum dislocation density was achieved after six DRECE passes, which were accompanied by the formation that is typical for low-strain structures. The increasing dislocation density, as well as grain refinement throughout DRECE deformation, resulted in an increase in the mechanical properties. Annealing of the as-deformed sample resulted in grain growth and strength reduction.

## 1. Introduction

Severe plastic deformation (SPD) processes are regarded as appropriate for the manufacturing of bulk samples having a nano or ultrafine-grained microstructure via imposing significant plastic strain (which is difficult to obtain using the conventional deformation technique). Several different SPD processing methods are now available i.e., HPT (high-pressure torsion), TE (twist extrusion), or ARB (accumulative roll bonding) [1,2,3]. However, in recent years, the ECAP (equal channel angular pressing) method attracted the greatest interest. In this procedure, the workpiece, in the form of a rod or bar having a circular cross-section, is pressed through a die that consists of two channels that meet at a predetermined angle: usually 90 degrees [4]. A significant limitation of conventional ECAP is the size of the work samples and process discontinuity. The work sample must be repeatedly reinserted in the die channel for several consecutive passes to impose high strain. This type of procedure from a practical point of view has a disadvantage of high labor input requirement during the deformation process, and it may be used easily only for fundamental laboratory investigations. The ECAP process is discontinuous and slow, which makes it impractical in industrial applications. Due to that, critical attention has been focused on the possibility of alternative processing procedures development that is continuous and precludes the need for removing and reinserting the work samples after every deformation cycle. Several attempts have been made to enhance the effectiveness of the SPD processes. For instance, continuous cyclic bending [5], repetitive corrugation and straightening (RCS) [6], accumulative roll bonding [7], ECAP conform [8], or dual rolls equal channel extrusion (DRECE) [9] have been used successfully to produce fine-grained microstructures on a large scale.

The DRECE method is a modification/combination of two commonly known SPD techniques: the DCAP method (dissimilar channel angular pressing) and ECAP-CONFORM (continuous equal-channel angular pressing), and it has been developed for forming metal sheets with maximum dimensions of 1000 × 60 × 2 mm. In this technique, the sample is inserted into the working space and then extruded through the roller without changing their dimension. The critical factor that influences the mechanical properties of the material after the DRECE process is a proper selection of the output geometry in the deformation zone–deformation angle (123°, 118° or 108°) [10]. When comparing the DRECE method to the other commonly known SPD techniques, we must remember that the strain accumulation after a single pass is not as high as i.e., in the ECAP process. Similar to the ECAP process, the repetitive deformations increase the accumulated strain, which affects the microstructure and thus changes the mechanical properties. The main advantage of the DRECE method is the length of the work specimen and the form of the batch material sheet. Such form of the batch material is typical i.e., for automotive industry applications.

The 5754 aluminum alloy used in this study is typically supplied to automotive manufacturers, where it is used for car body parts and door structural components. This is because of its unique properties, including moderate strength, high corrosion resistance, weldability, and easy forming characteristics [11]. These properties can be increased by severe plastic deformation processes by employing the grain refinement process [12,13]. However, conventional SPD processes provide only laboratory-scale samples, which cannot be applied industrially. Therefore, in this investigation, we focused on the DRECE method and its possible application to enhance the mechanical properties of the 5754 aluminum–magnesium alloy.

## 2. Materials and Methods

The investigated material was an aluminum–magnesium alloy 5754 that had the chemical composition given in Table 1.

**Figure 1:**
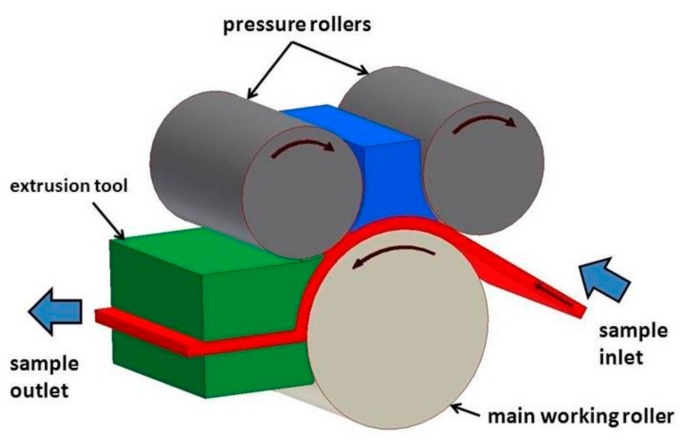
Principle of the dual rolls equal channel extrusion (DRECE) process.

The DRECE process with the principle is shown in Figure 1 was conducted at VŠB-Technical University in Ostrava in the Department of Mechanical Engineering. In this experiment, we set the deformation α angle to be 108°.

The 5754 alloy samples covered with a Gleit grease were subjected up to six DRECE passes without changing the sample orientation between subsequent passes. The pressure of the feed roller was set to 150 bar. The sample was processed with a constant deformation speed of 40 mm/min. To investigate the thermal stability of the obtained microstructure, the sample was subjected to isothermal annealing at 150 °C, 180 °C, 200 °C, 250 °C, 300 °C, and 350 °C respectively for 30 min after six DRECE passes.

The microstructures were examined using a light microscope Axio Observer Z1. To reveal the grain size and morphology, the samples were mechanically ground and polished and then electrolytically etched using Barker’s reagent.

To study the microstructural evolution in a greater detail, the 5754 aluminum alloy samples were twin-jet electropolished in an electrolyte containing 20% nitric acid and 80% methanol at a temperature of −30 °C and 20 V for 15 s. Then, the grain structure was recorded by orientation imaging microscopy (OIM) using the electron backscattered diffraction (EBSD) technique integrated with a Zeiss Supra 35 Scanning Electron Microscope controlled and analyzed using OIM software (EDAX, Inc., Mahwah, NJ, USA). An area 60 × 60 μm was scanned on the normal direction plane, and the step size was taken to be 0.2 μm in all scans. A neighbor orientation correlation data cleaning process (level 4), followed by a grain confidence index (CI) standarization cleanup with a grain tolerance angle threshold set to 2°, was used. After the data cleaning, a coincidence index filter of 0.05 was used to remove the points that were not indexed correctly.

X-ray diffraction analysis was carried out on the electropolished sections of the samples by employing a PANalytical X’Pert Pro diffraction system (Malvern Panalytical Ltd., Royston, UK) equipped with a CuKα radiation source, 30 kV, and 30 mA with a scan rate of 0.005°/s. The average crystallite size was calculated from the four Bragg reflection peaks of fcc-Al: (111), (200), (220), and (311) using the Scherrer and Wilson equation [14].

(1) $$D_{v}=\frac{0.9\lambda }{Bcos\theta_{B}}$$

where *D_v_* is the effective crystallite size, λ is the X-ray wavelength, *θ* is the Bragg angle, and *B* is the line broadening. The microstrain was calculated using the Williamson Hall equation [15].

(2) $$Bcos\theta =\left[\frac{0.9\lambda }{D_{v}}\right]+\left[4\varepsilon sin\theta \right]$$

where ε is the root mean square of microstrain. According to the Rietveld method, the dislocation density can be estimated from the following equations:

(3) $$\rho ={\left(\rho_{d}\cdot \rho_{s}\right)}^{1/2}$$

(4) $$\rho_{d}=3/D_{v}^{2}$$

(5) $$\rho_{s}=\varepsilon^{2}/b^{2}$$

where ρ_d_ and ρ_s_ are the dislocation density due to domains and the dislocation density due to the microstructure, respectively.

Finally, the microhardness (HV) of the specimens were measured along the thickness by a microhardness tester Future-Tech FM-ARS under a load of 300 g for 15 s. Tensile tests were carried out using Zwick Z100 equipment (ZwickRoell GmbH & Co.KG, Ulm, Germany) with a strain rate of 6.7 × 10^−4^ s^−1^.

## 3. Results and Discussion

### 3.1. Initial Microstructure

**Figure 2:**
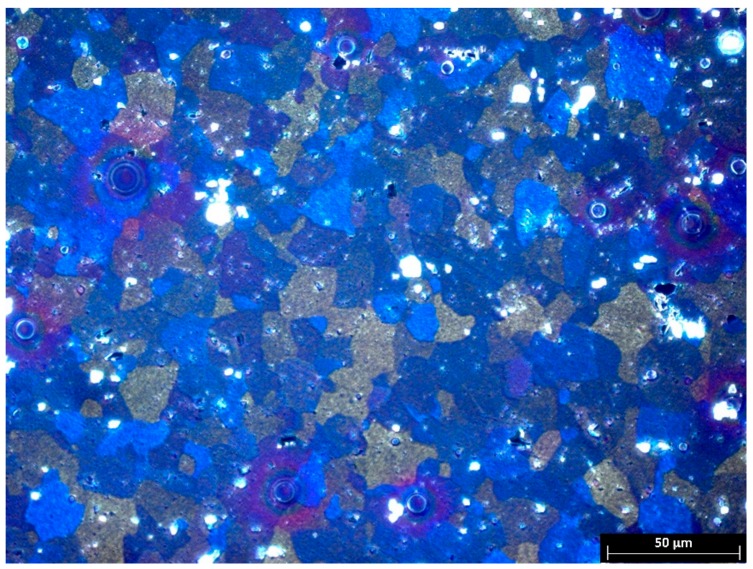
Microstructure of the 5754 aluminum alloy in an initial state.

Figure 2 is an optical micrograph of 5754 alloy in an unprocessed state. This figure shows a typical wrought microstructure built of equiaxed grains. According to Cabibbo [16], the 5754 alloy matrix consist of a Mg in Al solid solution with fine dispersoids of Mg_2_Si and Al_6_Mn phases.

### 3.2. Effect of DRECE on Dislocation Density

**Figure 3:**
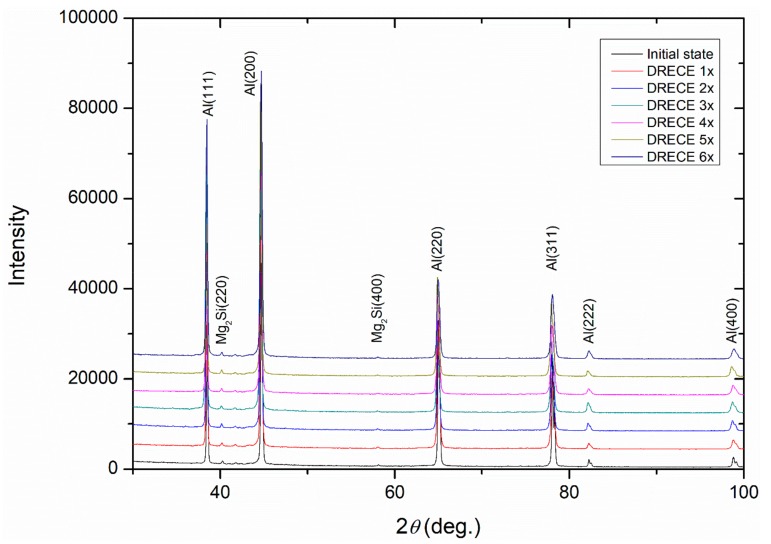
X-Ray diffraction profiles of 5754 aluminum alloy in different conditions.

Figure 3 shows the X-ray diffraction profiles of 5754 alloy samples in a different condition. As shown, the main reflections for the fcc Al (111), (200), (220), (311), and (400) were detected. In addition, two supplementary peaks at 2θ = 40.21° and 58.11 were identified to be characteristic of the Mg_2_Si phase. The diffraction peaks of the as-deformed samples are broadened, and their intensities change throughout the DRECE process. According to Ungar [17], this can be due to an increased amount of dislocations accumulated during severe plastic deformation and the small size of the diffracting grains Additionally, the peak shift expresses that the DRECE process causes a substantial amount of distortion in the lattice structure of Al5754 alloy. This can be related to long range stresses generated by severe plastic deformation.

The summarized results of the structural parameters obtained from XRD analysis are presented in Table 2. Throughout DRECE processing, the average domain size parameter decreased from ~35 nm in an initial state to ~29 nm after six DRECE passes. In addition, after DRECE processing, the dislocation density increased from 3.46 × 10^14^ m^−2^ in the unprocessed state to 6.47 × 10^14^ m^−2^ after the first pass. Then, the dislocation density showed a steady decrease with an increasing number of passes, indicating the annihilation of dislocations despite increased strain accumulation [18], reaching a saturation level of 7.55 × 10^14^ m^−2^ after six DRECE passes. This result is in accordance with the results of other works for similar Al–Mg alloys. Dinkerl [19] has documented that the dislocation density of AlMg2 alloy was calculated by XRD to be ~5 × 10^14^ m^−2^~3 × 10^14^ m^−2^ and 12 ECAP passes, respectively. According to Liu [20], the dislocation density of an AA5182 alloy after five turns of the HPT process increased to 12.8 × 10^14^ m^−2^.

### 3.3. Evolution of Microstructure

**Figure 4:**
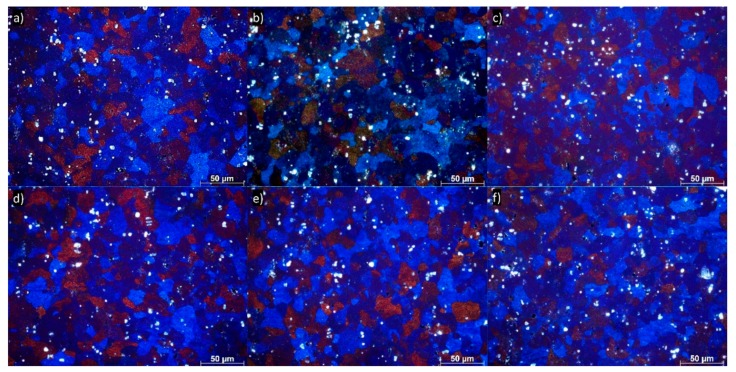
Examples of grain structures of 5754 aluminum alloy subjected to (**a**) one DRECE pass, (**b**) two DRECE passes, (**c**) three DRECE passes, (**d**) four DRECE passes, (**e**) five DRECE passes, (**f**) six DRECE passes.

Figure 4a–f shows an optical micrograph (longitudinal plane) of 5754 aluminum alloy subjected up to six DRECE passes. As compared with the initial state of the microstructure (Figure 2), the grain size is not reduced significantly. Areas of different crystallographic orientations inside individual grains are visible. For the micrographs shown, it appears that the increase in the number of DRECE passes is not accompanied by noticeable changes in the microstructure. Throughout subsequent passes, the grains remain equiaxed and almost constant in size.

**Figure 5:**
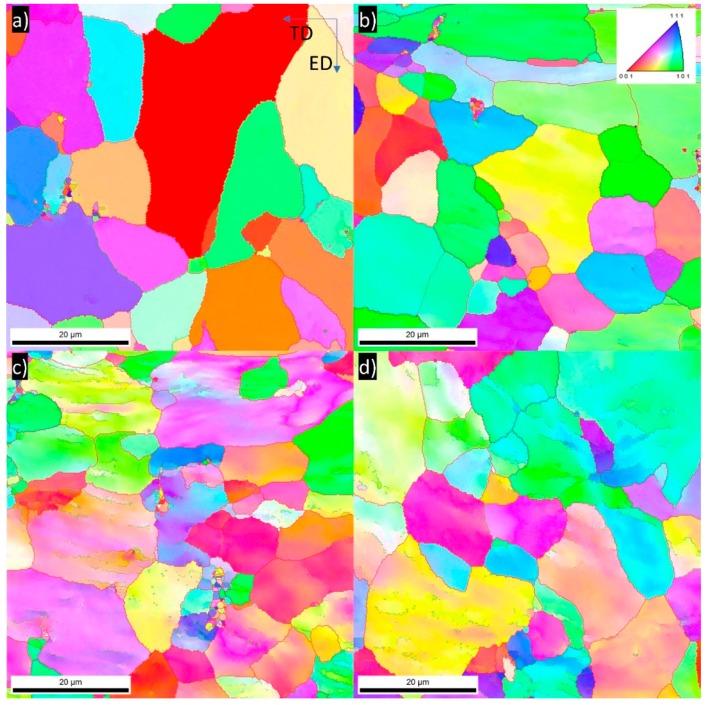
Electron backscattered diffraction (EBSD) inverse pole figure (IPF) maps of 5754 aluminum alloy (**a**) initial state, (**b**) one DRECE pass, (**c**) four DRECE passes, (**d**) six DRECE passes.

Figure 5 shows the EBSD colored inverse pole figure (IPF) maps of the 5754 aluminum alloy samples in an initial state and subjected to one, four, or six DRECE passes, respectively. In these microstructures, the grain color/shade corresponds to the individual grain orientation denoted by the unit triangle legend in Figure 5b. The red lines indicate the locations of high-angle grain boundaries (θ >15°), while green lines indicate the locations of low-angle grain boundaries (3°> θ >15°). The IPF image of the initial state sample (Figure 5a) demonstrates that the microstructure is composed of equiaxed grains. The measured average intercept length prior deformation is ~7.916 μm. Figure 5b shows the microstructure after the first DRECE pass. According to the unit triangle legend used, several orientations can be observed even in the grain interiors. This indicates the variation in crystal orientation in the grain interiors at the early stages of deformation. The average intercept length remains almost unchanged: ~7.862 μm. Figure 5c shows the effect of four DRECE passes. In this condition, the grain interiors are covered with many elongated bands of cells, having a low-angle grain boundary misorientation. These deformation bands are almost parallel to the TD direction. The measured average intercept length decreased to ~6.872 μm. Figure 5d shows the microstructure of the sample subjected to six DRECE passes. In this state, the microstructure consists of grains covered with deformation bands having a low angle misorientation. This type of microstructure is typical for aluminum alloys subjected to low strains [21,22]. The measured average intercept length after six DRECE passes slightly increases to ~6.958 μm.

**Figure 6:**
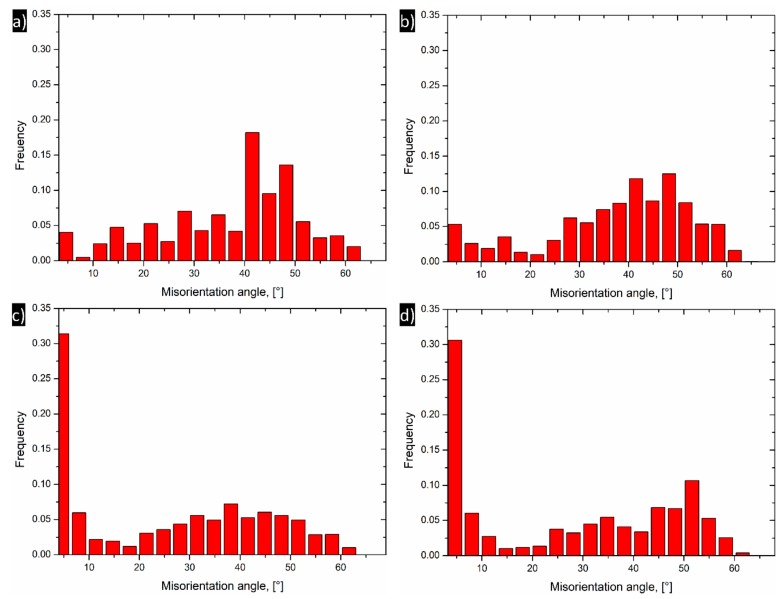
Misorientation distribution of the 5754 alloy samples (**a**) initial state, (**b**) one DRECE pass, (**c**) four DRECE passes, (**d**) six DRECE passes.

Detailed extracted values of the fraction of the grain boundaries are presented in Figure 6 and Table 3. In the initial state, the fraction of HAGBs is very high ~88.4% and slightly decreases after the first DRECE pass to ~86.6%. Along with the increase in the number of deformation passes, the fraction of HAGBs decreases. After four DRECE passes, approximately 58.6% of the grains have a grain boundary misorientation of more than 15°, indicating HAG (high-angled grains). Finally, the opposite trend is visible. After six DRECE passes, the fraction of HAGBs (high-angle grain boundaries) slightly increases to 59.6%.

### 3.4. Mechanical Properties after DRECE Process

**Figure 7:**
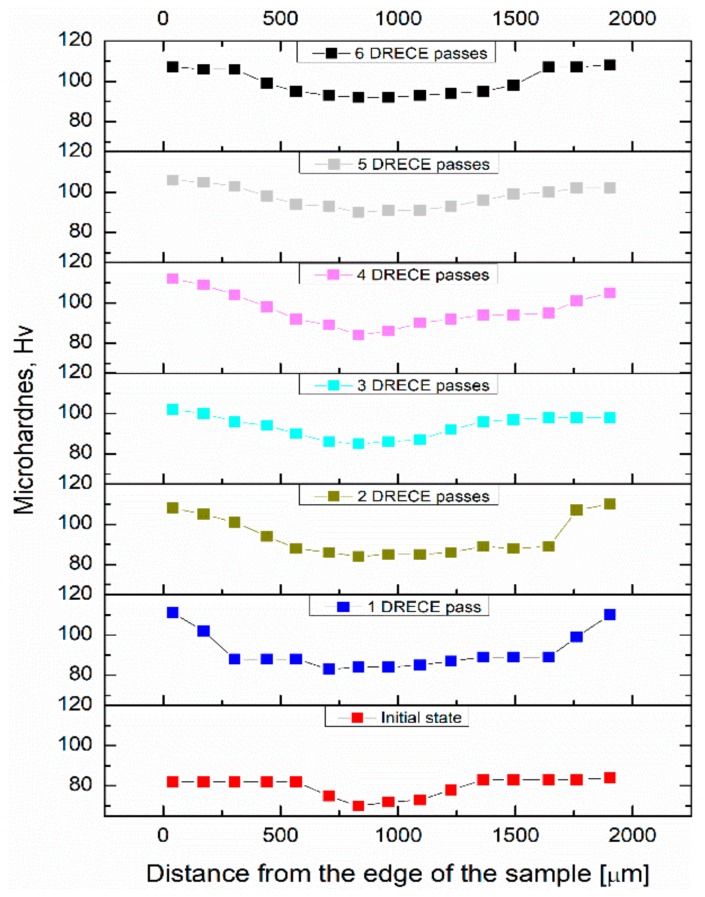
Hardness distribution along the sheet thickness of the 5754 aluminum alloy samples.

Figure 7 presents the indentation Vickers microhardness for an initial state and DRECE processed 5754 alloy samples taken across the thickness of the samples. Additionally, a summary of mechanical properties as a function of DRECE passes is given in Table 4. The observed high hardness gradient between the surface and center of the initial state sample is due to the inhomogeneity of deformation during rolling. The measured mean microhardness value is ~79 Hv. The hardness of the sheet increases throughout DRECE deformation. After the first DRECE pass, this value raises to about ~92 Hv. In addition, in this state, the hardness gradient between the surface and center increases—the hardness values lie in the range of 82–111 Hv. The observed hardness increase is accompanied by a dislocation density growth, as presented in Table 2. Along with increasing strain accumulation, the hardness values slightly increase, reaching a maximal value of ~99 Hv after six passes. It is worth mentioning that the hardness gradient between the surface and center of the samples decreases throughout DRECE deformation—the hardness values lie in the range of 92–108 Hv, indicating that the non-homogeneous hardness distribution (non-uniform deformation) in the as-rolled initial sample remains in the DRECE processed samples. This may be attributed to the nature of the rolling process, in which the imposed strain is higher near the surface, while a lower amount of deformation is applied at the center.

The mechanical properties of 5754 alloy after the DRECE process were also tested in a tensile test at room temperature, as shown in Table 4. It is clear that both yield and tensile strength increase throughout DRECE deformation. For the initial state, the mean yield strength value is 112.3, and this value increases rapidly to 184.7 MPa after the first pass. Then, it rises slightly to 198.2 MPa after six passes. This means that the work-hardening rate reduces with the strain increase. However, the observed strength improvement, which was lower than that with the ECAP method [23,24], was achieved at the expense of ductility, which decreased from 30.1% to about 13.2% after the first DRECE pass; then with an increase in strain accumulation, it decreased to 8.4% after the six DRECE passes. Such changes in strength and ductility are typical for severely deformed metals, as proved in numerous research papers [4,16,25,26].

### 3.5. Annealing Response of the As-Deformed 5745 Alloy

After the DRECE deformation, a thermodynamically unstable microstructure containing lattice defects and subgrain boundaries was produced. This distorted microstructure increased the mechanical properties. To allow further work operation such as shaping, stamping, or forming, the as-deformed alloy must be softened by annealing. The microstructural changes that occur upon annealing are commonly described with reference to recovery processes, the nucleation of new grains, and their growth. The recovery process reduces the stored energy, but it causes only partial mechanical strength reduction, since the dislocation structure is not rearranged into a stable state.

A further microstructural restoration process—recrystallization—may occur in which a new set of defect-free grains nucleate within the as-deformed microstructure. Although the recrystallization removes some amount of stored dislocations, the material still contains a large fraction of grain/subgrain boundaries, which are thermodynamically unstable. Further annealing causes grain growth, which goes along with the formation of new (defect-free) grains in a lower energy configuration.

**Figure 8:**
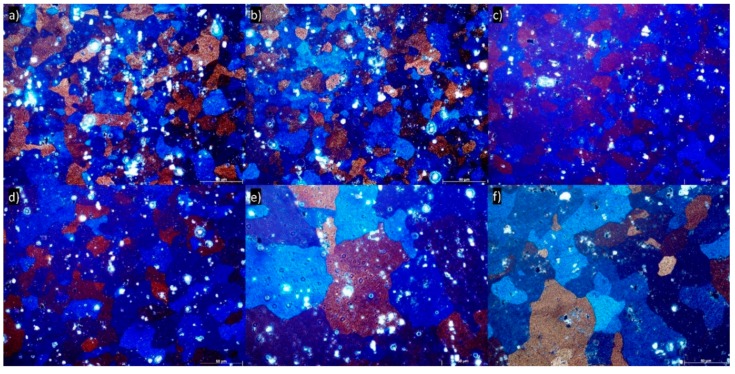
Examples of grain structures of 5754 aluminum alloy subjected to annealing for 30 min (**a**) 150 °C, (**b**) 180 °C, (**c**) 200 °C, (**d**) 250 °C, (**e**) 300 °C, and (**f**) 350 °C.

Light microscopy characterization of the grain microstructure of the 5754 alloy annealed at 150 °C, 180 °C, 200 °C, 250 °C, 300 °C, and 350 °C is shown in Figure 8. It is apparent that during annealing at 150 °C, the process of recovery dominates. This is accompanied by a substantial dislocation density decrease from 7.55 × 10^14^ m^−2^ in the as-deformed condition to 4.68 × 10^14^ m^−2^, as shown in Table 5. During annealing at 180–300 °C, the entire microstructure undergoes continuous recrystallization followed by grain growth (Figure 8b–e). The first effect of annealing is visible in the sample annealed at 180 °C (Figure 8b). In this condition, small recrystallized grains can be observed in the microstructure. The formation of the new grains is accompanied by a continuous dislocation density decrease to 4.44 × 10^14^ m^−2^. The DRECE processed structure changes completely into coarse-grained and is approximately equiaxed with an increase in annealing temperature, obviously, as illustrated in Figure 8e. This is followed by a gradual dislocation density decrease to 2.05 × 10^14^ m^−2^. It is worth mentioning that for the sample annealed at 350 °C, the microstructure exhibit characteristics of both uniform coarsening and, in several places, of discontinuous recrystallization (Figure 8f). Thus, the obtained microstructure is bimodal, and the formation of the bimodal microstructure is accompanied by a slight dislocation density growth to 3.18 × 10^14^ m^−2^. This is due to the presence of the new annealed microstructure that gives rise to compressive stress fields to the surrounding small-sized grains, resulting in a relatively lattice distortion and enhancing the dislocation density.

**Figure 9:**
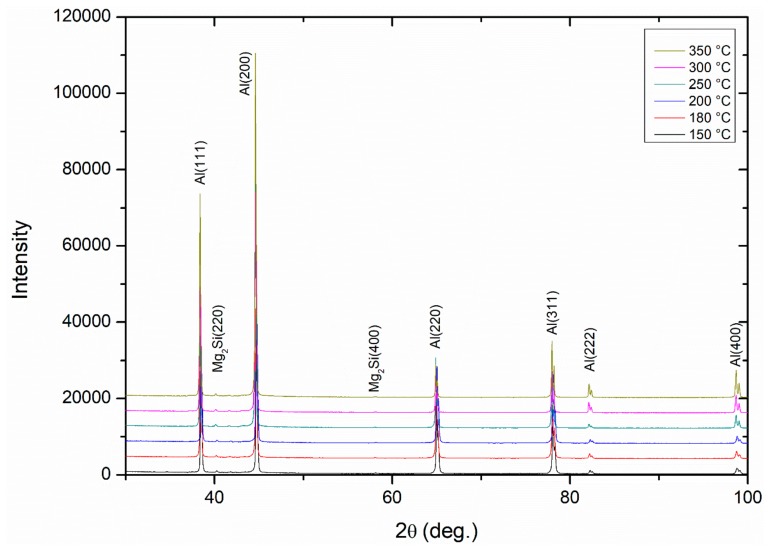
X-Ray diffraction profiles of DRECE processed and annealed 5754 aluminum alloy.

Figure 9 shows the X-ray diffraction profiles of the DRECE processed and annealed 5754 alloy samples. As shown, only the main reflections for the fcc Al (111), (200), (220), (311), and (400) and Mg_2_Si (220) and (400) phases were detected. The diffraction peaks are slightly broadened, and their intensities change depending on the annealing temperature. The intensity and width of the peaks in an XRD pattern can be correlated to the level of residual stresses and dislocation density, which changes after annealing.

The evolution of Vicker’s microhardness of the isothermally annealed 5754 alloy samples is listed in Table 6. The obtained hardness measurements results are in good agreement with the observed microstructure evolution as well as with structural parameters calculated by XRD.

It is evident, as shown in Table 6, that the hardness decreases rapidly from an initial value of ~99.5 HV after six DRECE pass samples to 81.5 HV after 30 min of isothermal annealing at 150 °C. It is due to the ongoing recovery processes and is accompanied by a rapid dislocation density decrease from 7.55 × 10^14^ m^−1^ to 4.68 × 10^14^ m^−1^. In this condition, the changes in the grain size are almost indistinguishable (Figure 8), and there is no evidence of the recrystallization processes. With an increase in an isothermal annealing temperature up to 180–200 °C, hardness decreases slightly to ~77 HV. This is accompanied by only a slight dislocation density decrease to 4.16 × 10^14^. In addition, the microstructure looks quite similar—there is still no evidence of the recrystallization process, see Figure 8a–c. The first evidence of the recrystallization and grain growth can be observed in the sample isothermally annealed at 250 °C; see Figure 8d. This phenomenon is accompanied by a dislocation density reduction and hardness decrease to ~61 HV. Annealing at 300 °C causes a more significant grain growth, as shown in Figure 8e, as well as the greatest dislocation density drop to 2.05 × 10^14^. However, despite the observed microstructural changes, the hardness remains almost unchanged. It should be pointed out that when the material is subjected to isothermal annealing at 350 °C, a slower softening rate is observed. In addition, the obtained microstructure is different: bimodal. This occurs because some grains have a much faster growth rate than others. Such a bimodal microstructure is inhomogeneous and is built of recrystallized and recovered grains and volumes with an unchanged distorted thermodynamically metastable structure; therefore, it combines the effects of the larger grains, which cause softening and the ultrafine grains that increase the mechanical properties.

In general, the difference in softening behavior reported in this study as a function of increasing annealing temperature is in line with competitive recovery–recrystallization kinetics, where a lower isothermal annealing temperature results in a gradual hardness decrease and resembled softening due to recovery, while at higher temperatures where recrystallization processes arise quickly, softening is faster.

## 4. Conclusions

An investigation was conducted to study the evolution of microstructure and hardness in bulk aluminum 5754 samples processed by DRECE. After DRECE, the samples were subjected to annealing in order to investigate the microstructure and mechanical properties. The main conclusions of this research are summarized below.

The grain boundary maps for the EBSD data revealed that the fraction of low-angle grain boundaries (LAGBs) increases along with an increasing number of DRECE passes. The DRECE process introduces a network of LAGBs, which may transform into HAGBs with further processing, especially if new shear planes will be activated.

The DRECE process increases the hardness of the 5754 aluminum alloy of about 25% after six passes. It is presented also that the yield and tensile strength reached their maximum values of 198 MPa and 251.8 MPa, respectively, after six passes. The increment of the Vickers microhardness as well as tensile and yield strength can be related to increased dislocation density and grain refinement. 

The post-DRECE annealing of six passes of 5754 alloy samples at temperatures lower than 180 °C did not affect the microstructure significantly, indicating that recovery dominates. A continuous grain growth occurred at higher annealing temperatures (180–300 °C) due to the continuous recrystallization. A bimodal microstructure having a bimodal dislocation density distribution was obtained through the discontinuous recrystallization at 350 °C.

The hardness measurements of the annealed sample were consistent with the microstructural and XRD study. Grain coarsening led to a stepwise hardness decrease and resembled softening due to recovery before recrystallization. After annealing at the highest temperature (350 °C), the alloy microhardness significantly decreased to a value lower than that of the initial state alloy.

## Figures and Tables

**Table 1:** Chemical composition of the 5754 aluminum alloy.

Al	Mg	Si	Mn	Fe	Cr	Cu	Zn	Ti
Bal.	2.6–3.6	≤0.4	≤0.5	≤0.4	≤0.3	≤0.1	≤0.2	≤0.15

**Table 2:** Structural parameters of the 5754 aluminum alloy determined by X-ray diffraction peak analysis. *D_v_* = domain size; *ρ_d_* = dislocation density due to lattice microstrain; *ρ*_s_ = dislocation density due to size effect; *ρ* = total dislocation density.

Sample	D_v_(nm)	ρ (m^−2^)	ρ_d_ (m^−2^)	ρ (m^−2^)
Initial state	35	2.46∙× 10^15^	4.86∙× 10^13^	3.46 × 10^14^
1×	32	2.98∙× 10^15^	1.40∙× 10^14^	6.47 × 10^14^
2×	34	2.63∙× 10^15^	1.35 × 10^14^	5.96 × 10^14^
3×	31	3.13 × 10^15^	1.04∙× 10^14^	5.73 × 10^14^
4×	30	3.15 × 10^15^	1.67∙× 10^14^	7.25 × 10^14^
5×	32	2.84 × 10^15^	1.50∙× 10^14^	6.54 × 10^14^
6×	29	3.36 × 10^15^	1.69∙× 10^14^	7.55 × 10^14^

**Table 3:** Summarized results of the EBSD analysis.

	Grain Size (Intercept Lengths) [μm]	Fraction of Low Angle Grain Boundaries, %	Fraction of High Angle Grain Boundaries, %	Average Misorientation Angle, θ_AV_ [°]
Initial state	7.916	11.6	88.4	36.98
1×	7.862	13.4	86.6	37.76
4×	6.872	41.4	58.6	25.50
6×	6.958	40.4	59.6	27.27

**Table 4:** Summary of mechanical properties of 5754 alloy.

Condition	Average Vickers Microhardness	Yield Strength (MPa)	Tensile Strength (MPa)	Elongation, %
Initial state	79.6	112.3	216	30.1
1×	91.7	184.7	235.4	13.2
2×	93.6	188.1	239.5	10.6
3×	93.7	189.4	241.2	10.0
4×	96.3	193.3	246.1	9.1
5×	97.5	194.1	247.0	9.0
6×	99.5	198.2	251.8	8.4

**Table 5:** Structural parameters of the DRECE processed and annealed 5754 aluminum alloy determined by X-ray diffraction peak analysis. *D_v_* = domain size; *ρ_d_* = dislocation density due to lattice microstrain; *ρ*_s_ = dislocation density due to size effect; *ρ* = total dislocation density.

Annealing Temperature [°C]	D_v_ (nm)	ρ_s_ (m^−2^)	ρ_d_ (m^−2^)	ρ (m^−2^)
150	34	2.50 × 10^15^	8.75 × 10^13^	4.68 × 10^14^
180	36	2.31 × 10^15^	8.51 × 10^13^	4.44 × 10^14^
200	37	2.14 × 10^15^	8.06 × 10^13^	4.16 × 10^14^
250	43	1.61 × 10^15^	8.63 × 10^13^	3.73 × 10^14^
300	40	1.85 × 10^15^	2.27 × 10^13^	2.05 × 10^14^
350	47	1.35 × 10^15^	7.48 × 10^13^	3.18 × 10^14^

**Table 6:** Average hardness values of DRECE processed and annealed 5754 alloy.

Annealing Temperature [°C]	Average Vickers Microhardness
150	81.5
180	74.1
200	77.1
250	61.1
300	61.5
350	68.7
